# Frovatriptan versus zolmitriptan for the acute treatment of migraine with aura: a subgroup analysis of a double-blind, randomized, multicenter, Italian study

**DOI:** 10.1007/s10072-012-1043-8

**Published:** 2012-05-30

**Authors:** Vincenzo Tullo, Gianni Allais, Marcella Curone, Michel D. Ferrari, Stefano Omboni, Chiara Benedetto, Bruno Colombo, Dario Zava, Gennaro Bussone

**Affiliations:** 1 Departement of Neuroscience, National Neurological Institute Carlo Besta, Milan, Italy; 2 Department of Gynecology and Obstetrics, Women’s Headache Center, University of Turin, Via Ventimiglia 3, 10126 Turin, Italy; 3 Leiden Centre for Translational Neuroscience, Department of Neurology, Leiden University Medical Centre, Leiden, The Netherlands; 4 Italian Institute of Telemedicine, Varese, Italy; 5 Departement of Neurology, San Raffaele Hospital, Milan, Italy; 6 Istituto Lusofarmaco d’Italia, Milan, Italy

**Keywords:** Migraine with aura, Frovatriptan, Zolmitriptan

## Abstract

Migraine with aura affects ~20–30 % of migraineurs and it is much less common than migraine without aura. The aim of this study was to compare the efficacy of frovatriptan 2.5 mg and zolmitriptan 2.5 mg in the treatment of migraine with aura. Analysis was carried out in a subset of 18 subjects with migraine with aura (HIS criteria) out of the 107 enrolled in a multicenter, randomized, double-blind, cross-over study. According to the study design, each patient had to treat three episodes of migraine in no more than 3 months with one drug, before switching to the other treatment. The rate of pain-free episodes at 2 h was significantly (*p* < 0.05) larger under frovatriptan (45.8 %) than under zolmitriptan (16.7 %). Pain free at 4 h, pain relief at 2 and 4 h and recurrent episodes were similar between the two treatments, while sustained pain-free episode was significantly (*p* < 0.05) more frequent during frovatriptan treatment (33.3 vs. 8.3 % zolmitriptan). Our study suggests that frovatriptan is superior to zolmitriptan in the immediate treatment of patients with migraine with aura, and it is capable of maintaining its acute analgesic effect over 48 h.

## Introduction

Approximately 20–30 % of people suffering from migraine headaches perceive an aura, namely a transient visual, sensory, language, or motor disturbance signaling the imminent occurrence of a migraine attack [1].

Though migraine with aura shares frequently the same clinical features with respect to the headache of migraine without aura, nevertheless the auras can cause anxiety and distress in the patient, being particularly disabling [2].

Triptans are generally considered the most effective acute treatment for migraine [3, 4]. However, pharmacological trials usually include mixed population of patients or predominantly migraineurs without aura, and do not specifically address drug efficacy in patients with aura [5].

To bring new evidence supporting the efficacy of triptans also in patients with migraine with aura, we analyzed a subgroup of such patients, included in a large randomized, double-blind, cross-over, comparative study of frovatriptan versus zolmitriptan [7].

## Methods

### Study population

Male or female subjects, aged 18–65 years, with a current history of migraine with or without aura, according to IHS criteria, and with at least one migraine attack per month for 6 months prior to entering the study, were eligible for participation in the main study [6, 7].

Details on study design and inclusion and exclusion criteria are available elsewhere [6]. In this analysis migraineurs with aura were selected. This condition was defined according to IHS criteria as at least two migraine attacks with aura symptoms, consisting of visual and/or sensory and/or speech symptoms [7].

### Study design

The study had a multicenter, randomized, double blind, cross-over design and has been extensively described in a previous publication [6]. Briefly, each patient received frovatriptan 2.5 mg or zolmitriptan 2.5 mg in a randomized sequence. After treating a maximum of three episodes of migraine in no more than 3 months with the first treatment, the patient had to switch to the other treatment and asked to treat a maximum of three episodes of migraine in no more than 3 months with the second treatment.

The study involved three visits and each patient’s participation time in the study did not exceed 6 months from randomization. Subjects having no migraine episodes during one of the two observation periods were excluded from the study.

Randomization was done by blocks of 4. Blindness was ensured by the over-encapsulation technique, i.e., by inserting study drug tablets in capsules.

### Data analysis

The present analysis was carried out in the subgroup of patients with migraine with aura, who actually treated at least one attack in each treatment period. Study endpoints were [7] (a) pain-free episode at 2 and 4 h (absence of migraine 2 and 4 h after intake of one dose of study drug and without any rescue medication); (b) pain relief at 2 and 4 h (defined as a decrease in migraine intensity from severe or moderate to mild or none at 2 and 4 h); (c) recurrence (pain free at 2 h and headache of any severity returning within 48 h); and (d) sustained pain-free episode within 48 h (migraine attack which is pain free at 2 h, does not recur and does not require the use of rescue medication or a second study drug dose within 48 h).

Continuous variables were summarized by the calculation of average values and standard deviation (SD), while categorical variables by computing the absolute value and the frequency (as percentage). Endpoints were compared between groups by generalized estimating equation analysis. The level of statistical significance was set at 0.05.

## Results

The whole intention-to-treat population consisted of 107 patients, of whom 18 (16.8 %) had migraine with aura and 89 (83.2 %) had migraine without aura. No statistically significant difference was observed between the two subgroups of patients for baseline characteristics Table 1.

**Table 1:** Baseline demographic and clinical data of the migraine patients with and without aura of the ITT population

	Migraine with aura (*n* = 18)	Migraine without aura (*n* = 89)	*p*
Age (years, means ± SD)	38.3 ± 6.1	38.1 ± 10.5	NS
Females (*n*, %)	16 (88.9)	69 (77.5)	NS
Height (cm, means ± SD)	164.2 ± 5.7	166.2 ± 8.3	NS
Weight (kg, means ± SD)	61.1 ± 6.7	63.7 ± 12.2	NS
Age at onset of migraine (years, means ± SD)	16.5 ± 9.6	16.3 ± 5.7	NS
Migraine attack duration >2 days (*n*, %)	3 (16.7)	14 (15.7)	NS
MIDAS score (means ± SD)	21.6 ± 14.3	22.8 ± 16.1	NS
No use of triptans in the previous 3 months (*n*, %)	8 (44.4)	22 (24.7)	NS
Patients with moderate attacks (*n*, %)	11 (61.1)	49 (55.1)	NS
Patients with severe attacks (*n*, %)	7 (38.9)	40 (44.9)	NS

Data are shown as mean (±SD), or absolute (*n*) and relative frequency (%). *P* refers to the statistical significance of the between-group differences

In the 18 patients suffering from migraine with aura, a total of 48 headache attacks were reported: 24 treated with frovatriptan (7.9 % of overall 304 episodes treated with this drug) and 24 treated with zolmitriptan (8.0 % of the overall 299 episodes).

**Fig. 1:**
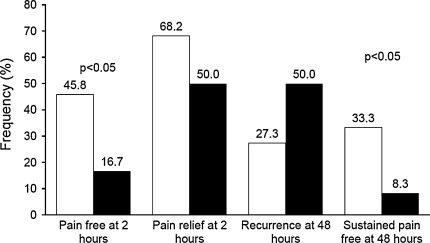
Rate of pain free at 2 h, pain relief at 2 h, recurrence at 48 h and sustained pain-free episode at 48 h in patients with migraine with aura treated with frovatriptan (*open bars*) or zolmitriptan (*full bars*). Data are shown as relative frequencies (%). *P* refers to the statistical significance of the between-treatment differences

As shown in Fig. 1, rate of pain-free episodes at 2 h was significantly (*p* < 0.05) larger under frovatriptan (45.8 %) than under zolmitriptan (16.7 %). Conversely, pain-free patients at 4 h were equally distributed between the two treatment groups (58.3 % for frovatriptan and zolmitriptan, *p* = NS). Proportions of patients with pain relief at 2 and 4 h and with recurrences did not significantly differ between frovatriptan (68.2, 72.7 and 27.3 %) and zolmitriptan (50.0, 63.6 and 50.0 %). Sustained pain-free episode was reported significantly (*p* < 0.05) more frequently during frovatriptan treatment (33.3 vs. 8.3 % zolmitriptan, Fig. 1).

## Discussion

In this study, we aimed at specifically comparing efficacy of two triptans in migraine patients with aura through a subgroup analysis of an original randomized, double-blind, cross-over study [6]. Treatment with frovatriptan 2.5 mg resulted in almost half of the patients free from pain at 2 h and more than one-third showing sustained pain-free episode at 48 h, with proportions larger than those observed under zolmitriptan. These results may have interesting clinical implications.

To our knowledge, this is the first direct head-to-head comparative study of two triptans in patients experiencing migraine with aura and strictly applying IHS criteria for definition of study endpoints. Although there are no previous studies specifically comparing the efficacy of frovatriptan and zolmitriptan in migraine with aura, our results are in line with those of previous randomized trials or meta-analyses which also included a small sample of migraineurs with aura and are also in line with the results obtained in migraineurs without aura [8–11].

In conclusion, results of our multicenter, randomized, double-blind trial support the indication of frovatriptan also for the management of the acute attack of migraine with aura. In this regard, frovatriptan seems to be superior to zolmitriptan, though future well-designed large-scale studies are needed to reinforce our observation, derived from a limited sample of subjects retrospectively analyzed.

## Appendix: List of study sites

Coordinator: G. Bussone (Milano).

Investigators: M. Gionco (Torino), A. Aguggia (Novi Ligure), B. Colombo (Milano), M. Turla (Esine), F. Perini (Vicenza), A. Ganga (Sassari), E. Agostoni (Lecco), C. Narbone (Messina), A. Moschiano (Merate), M. Vacca (Cagliari), M. Bartolini (Ancona), A. Ambrosini (Pozzilli), R. De Simone (Napoli), V. Petretta (Avellino), F. D’Onofrio (Avellino), D. Pezzola (Istituto Lusofarmaco d’Italia, Milano), G. Reggiardo (Biostatistical Unit, Mediservice, Milano), F. Sacchi (Clinical Unit, Mediservice, Milano).
